# Supplementary material for: Hepatic Hedgehog Signaling Participates in the Crosstalk between Liver and Adipose Tissue in Mice by Regulating FGF21
Source: Cells. 2022 May 18;11(10):1680. doi: 10.3390/cells11101680 (PMC9139566; doi:10.3390/cells11101680)
Supplement: Supplementary file 1 [file cells-11-01680-s001.zip › cells-1713642-supplementary.pdf]

Supplementary Material

Hepatic hedgehog signaling participates in the cross-talk between liver and adipose tissue in mice by regulating FGF21

Fritzi Ott[1,2#] and Christiane Körner[1,2#], Kim Werner[2,], Martin Gericke[3], Ines Liebscher[1], Donald Lobsien[4,5], Silvia Radrezza[6], Andrej Shevchenko[6], Ute Hofmann[7], Jürgen Kratzsch[8], Rolf Gebhardt[1], Thomas Berg[2], Madlen Matz-Soja[1,2*]

1 Rudolf-Schönheimer Institute for Biochemistry, Faculty of Medicine, Leipzig University, 04103 Leipzig, Germany; fritzi.ott@medizin.uni-leipzig.de; christiane.koerner@medizin.uni-leizig.de; kimfabienne66@gmail.com; ivs.gebh@t-online.de
2 Division of Hepatology, Clinic and Polyclinic for Oncology, Gastroenterology, Hepatology, Infectious Diseases, and Pneumology, University Hospital Leipzig, 04103 Leipzig, Germany; thomas.berg@medizin.uni-leipzig.de
3 Institute for Anatomy, Faculty of Medicine, Leipzig University, 04103 Leipzig, Germany; martin.gericke@medizin.uni-leipzig.de
4 Institute for Diagnostic and Interventional Radiology and Neuroradiology, Helios Clinic Erfurt; 99089 Erfurt, Germany; donald.lobsien@helios-gesundheit.de
5 Institute for Neuroradiology, University Hospital Leipzig, 04103 Leipzig, Germany
6 Max Planck Institute of Molecular Cell Biology and Genetics, 01307 Dresden, Germany; radrezza@mpi-cbg.de; shevchenko@mpi-cbg.de
7 Dr. Margarete Fischer-Bosch Institute of Clinical Pharmacology, University of Tübingen, 70376 Stuttgart, Germany; ute.hofmann@ikp-stuttgart.de
8 Institute of Laboratory Medicine, Clinical Chemistry and Molecular Diagnostics, Faculty of Medicine; Leipzig University, 04103 Leipzig, Germany; juergen.kratzsch@medizin.uni-leipzig.de
# These authors contributed equally to the manuscript.
* Correspondence: Madlen Matz-Soja madlen.matz-soja@medizin.uni-leipzig.de

Supplementary Figures

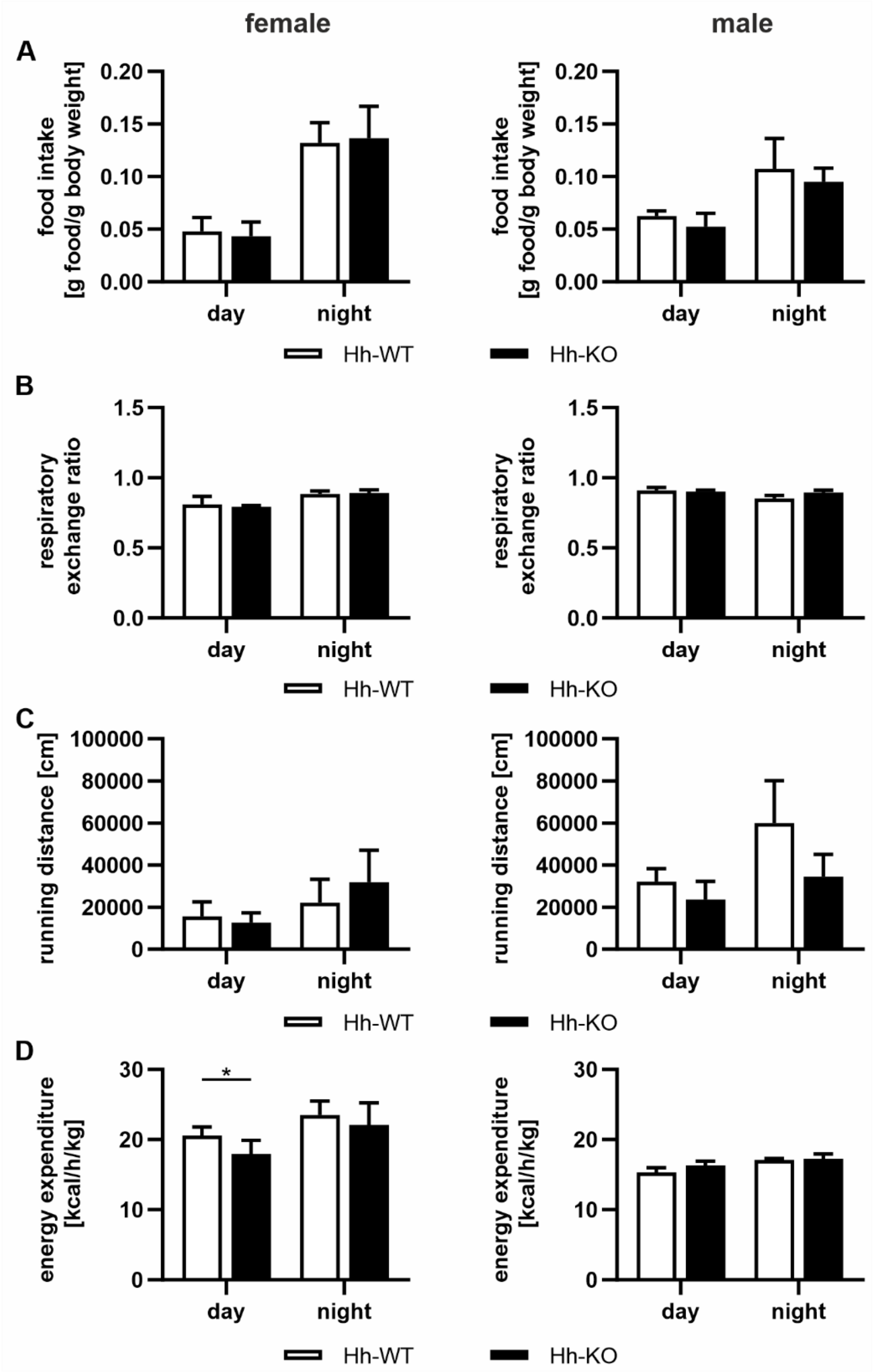

Figure S1. Metabolic cage analysis. Female and male Hh-WT and Hh-KO mice were fed a chow diet and examined in a metabolic cage with respect to (**A**) food consumption, (**B**) respiratory exchange ratio, (**C**) running distance and (**D**) energy expenditure. n = 4-6, multiple unpaired t tests with p values * ≤ 0.05.

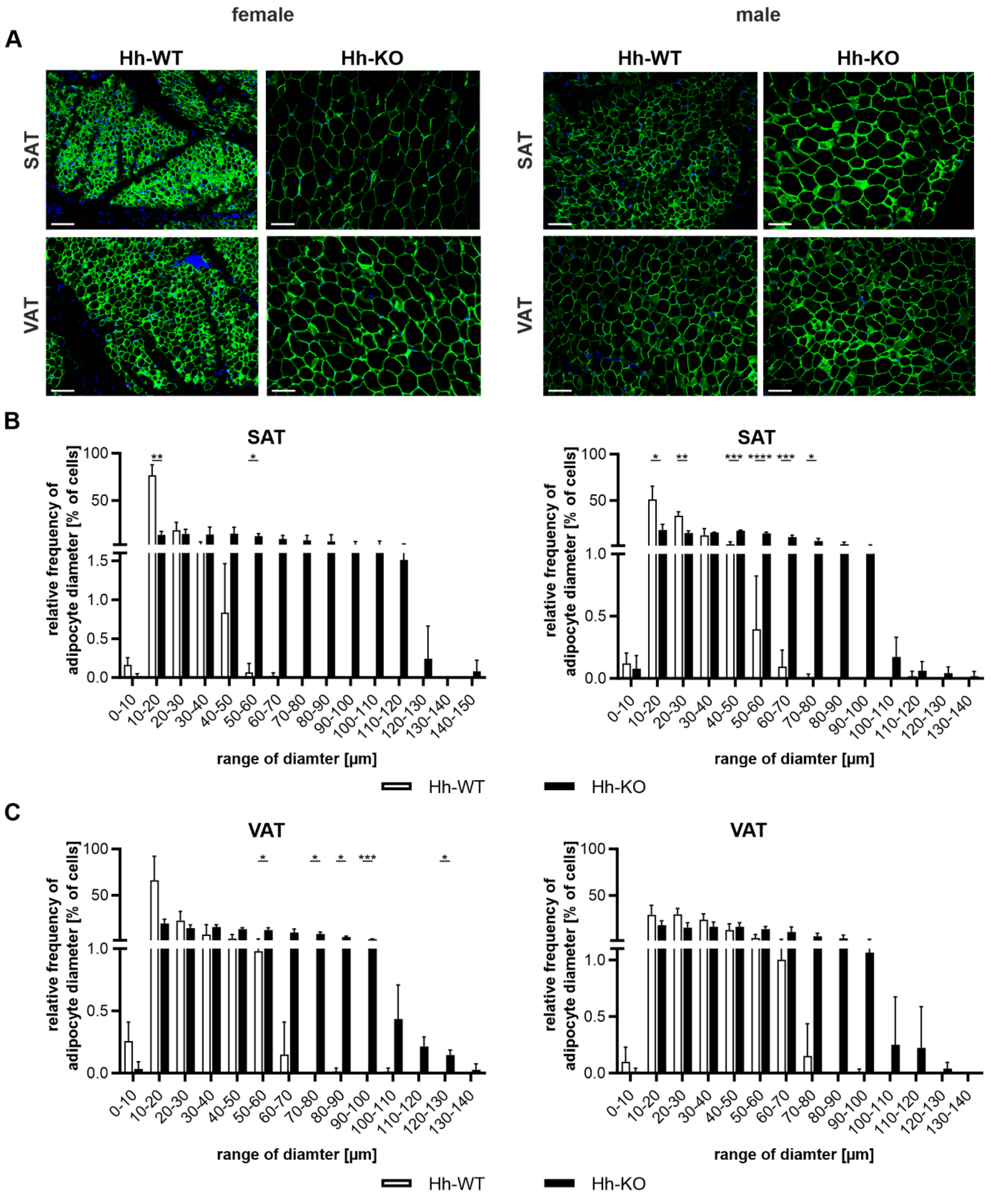

Figure S2. Quantification of adipocyte size. (**A**) Perilipin staining in SAT and VAT in male and female Hh-WT and Hh-KO mice. Scale bars indicate 100 µm. (**B-C**) Relative frequency of adipocyte diameter in percent of cells in (**B**) SAT and (**C**) VAT from male and female Hh-WT and Hh-KO mice. n = 3-5, multiple unpaired t tests with p values * ≤ 0.05; ** ≤ 0.01; *** ≤ 0.001; **** ≤ 0.0001.

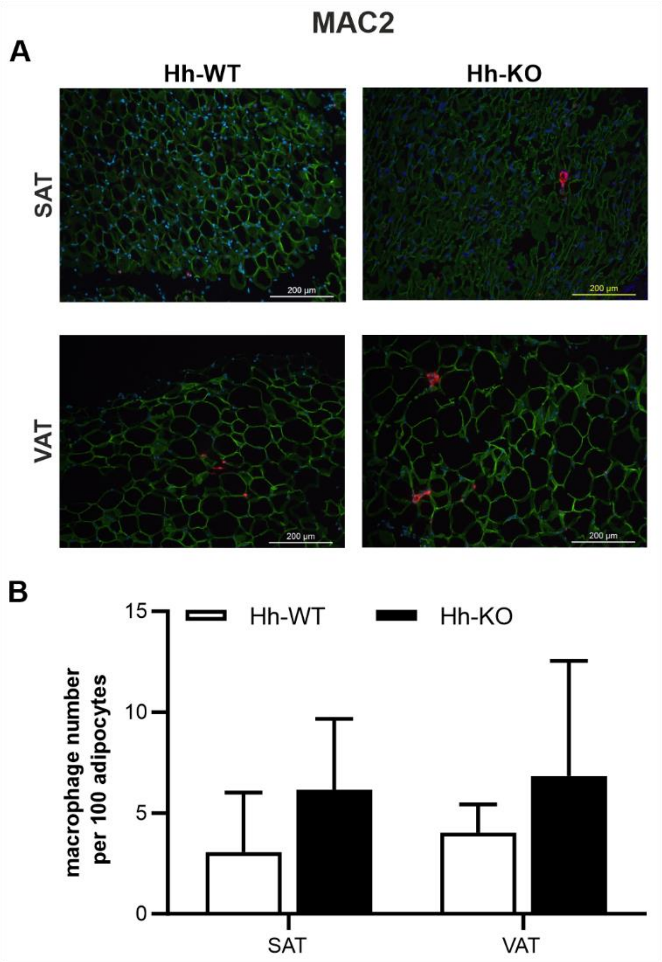

Figure S3. Characterization of inflammation in adipose tissue. (**A**) MAC-2 staining in SAT and VAT from male Hh-WT and Hh-KO mice. Scale bars indicate 200 μm. (**B**) Macrophage number per 100 adipocytes in SAT and VAT of male Hh-WT and Hh-KO mice. n = 4-5, multiple unpaired t tests.

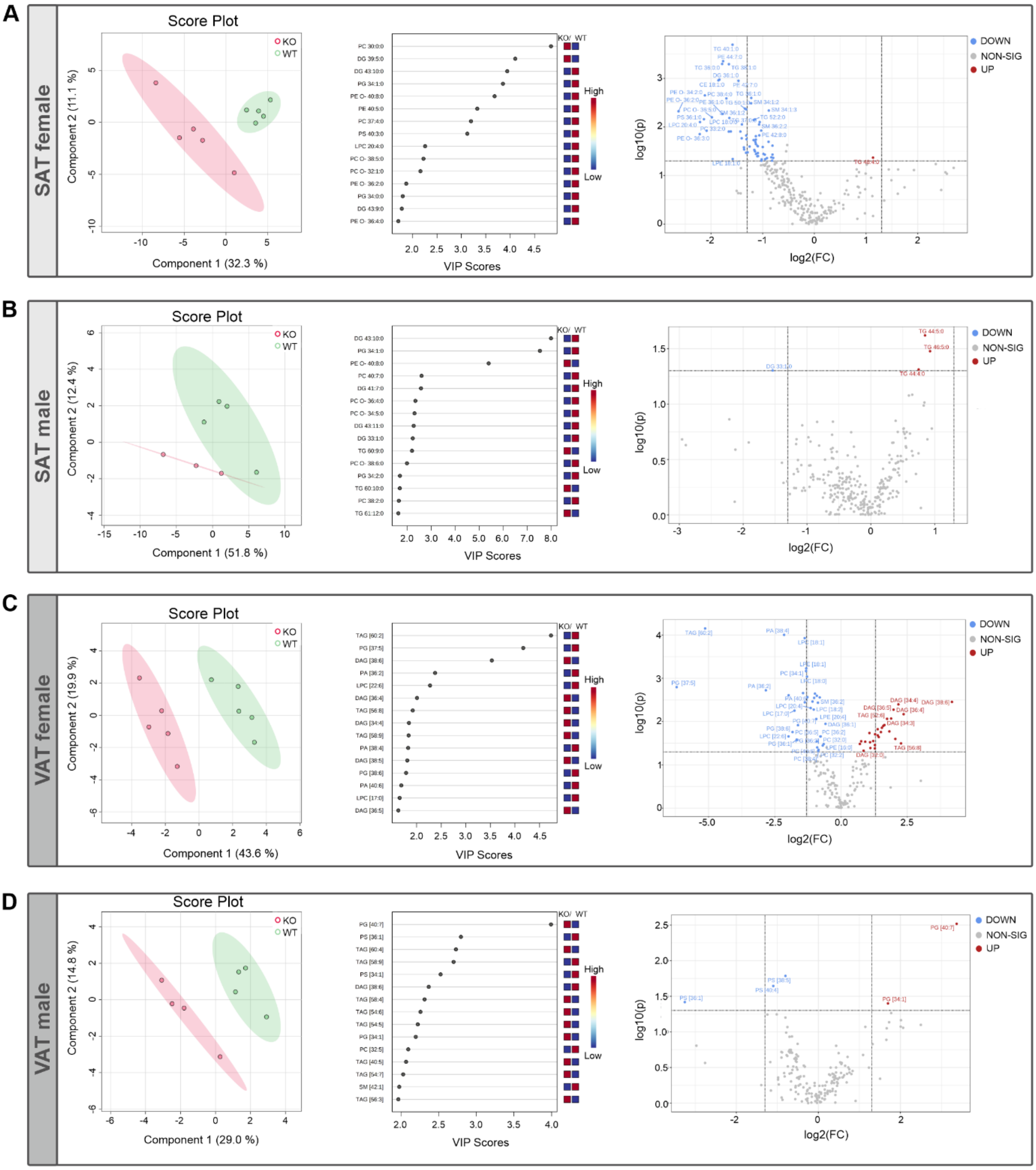

Figure S4. Lipidomic profiles of adipose tissue. Shotgun lipidomic analyses were performed on SAT and VAT from male and female Hh-WT and Hh-KO mice; n = 3-5. (**A-D**) Partial least squares discriminant analysis (PLS-DA) score plots generated from each genotype and sex. Variable of importance in projection (VIP) scores of each lipid species used in the PLS-DA. Lipids contributing meaningfully to the PLS-DA model with a PC1 VIP score >1.0 constituting 20% of the lipids tested (inset pie-chart). Volcano plot showing differentially expressed lipids between different libraries. A p value < 0.05 was used as the threshold to judge the significance of differences in lipid levels. Lipid species are marked as upregulated (red dots), downregulated (blue dots) or nonsignificant (gray dots).

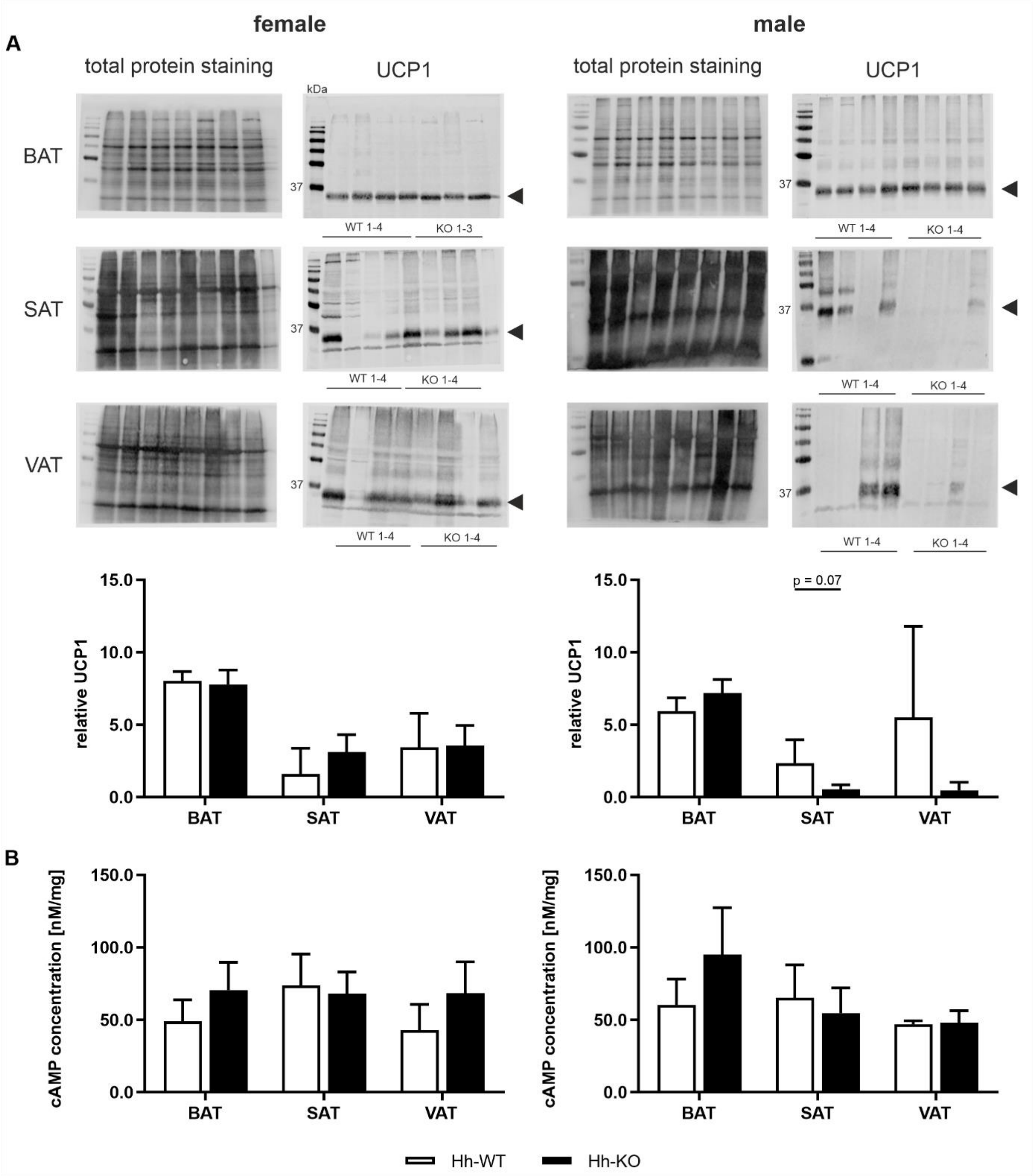

Figure S5. Quantitative western blot analysis of UCP1 and cAMP levels in adipose tissue. (**A**) Total protein staining and UCP1 western blot analysis in BAT, SAT and VAT from female and male Hh-WT and Hh-KO mice. Total protein staining was used for normalization of the UCP1 staining signal. (**B**) Basal cAMP levels in BAT, SAT and VAT in female and male Hh-WT and Hh-KO mice. n = 3-4, multiple unpaired t tests.

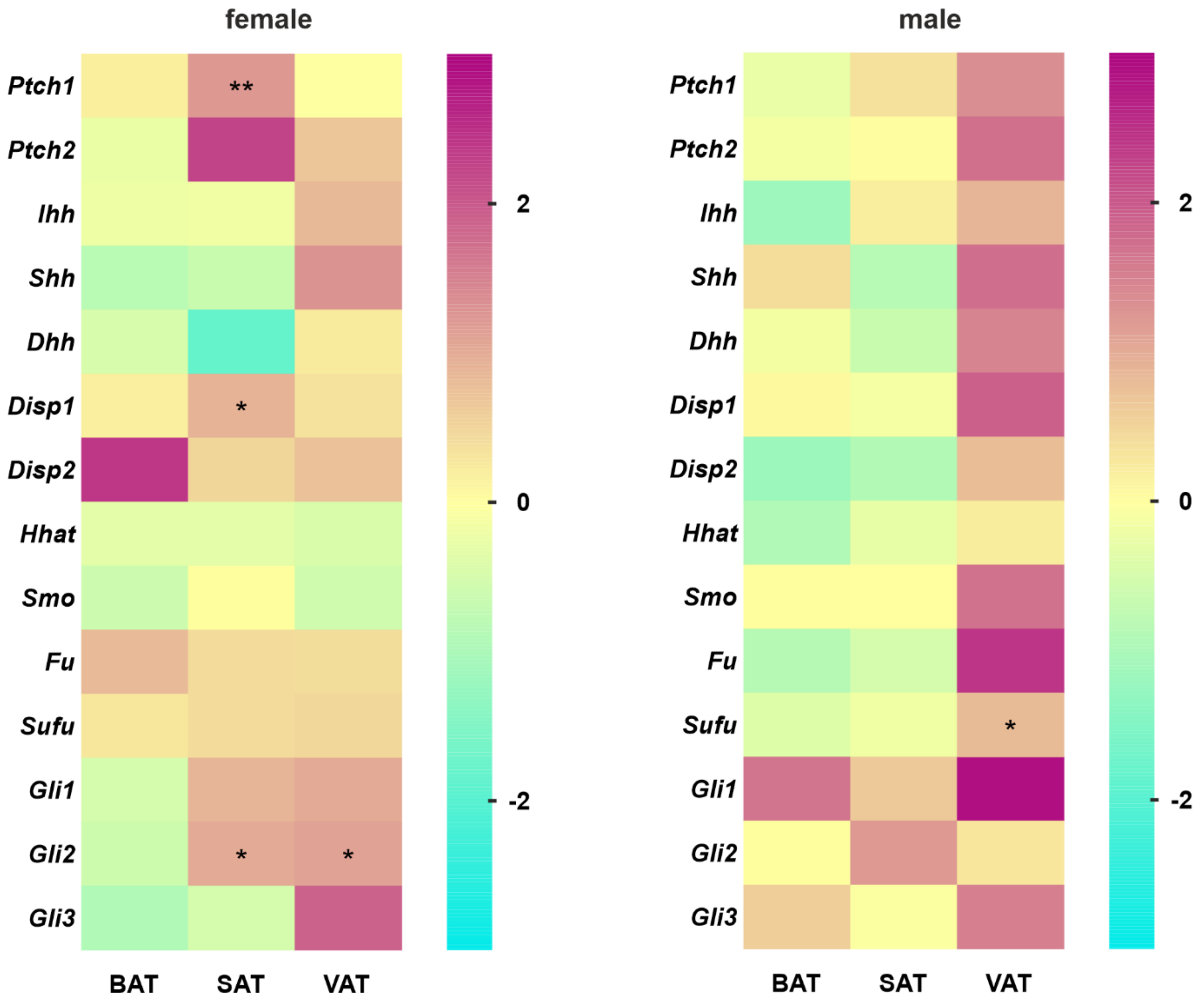

Figure S6. Hedgehog signaling-related gene expression profiles. Expression was quantified by qPCR in female and male Hh adipose tissue. Values are plotted as $\log_2$ fold changes, n = 4-6, multiple unpaired t tests with p values * ≤ 0.05, ** ≤ 0.01. Numerical values are available in Table S8.

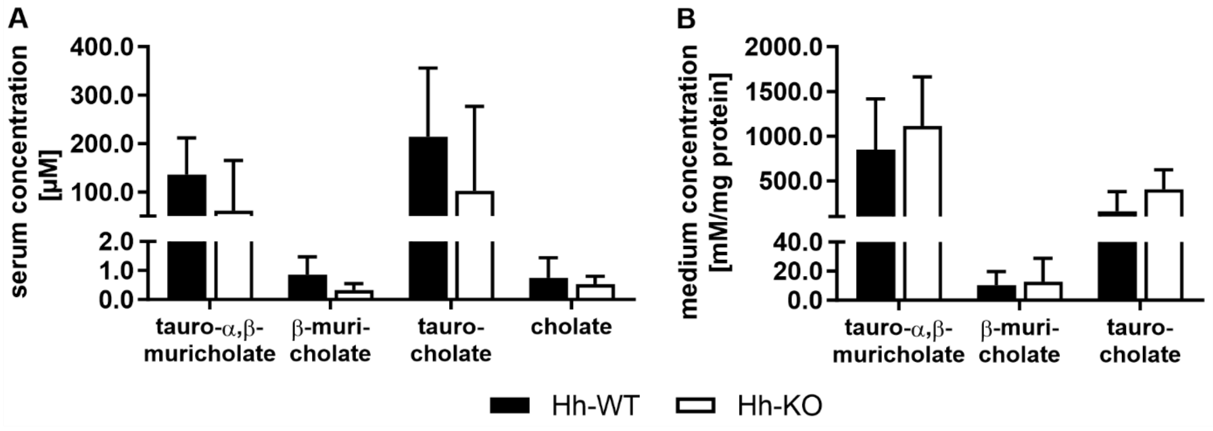

Figure S7. Bile acid concentrations. Quantification of bile acids in (**A**) serum and (**B**) hepatocyte supernatants from Hh-WT and Hh-KO mice. n = 3-6, multiple unpaired t tests.

Supplementary Tables

Table S1. Forward and reverse primer sequences used for qPCR.

gene name	forward primer	reverse primer
Agpat	AAGTATAACCCCCAGTTCGGC	TACCACACGTCGCAGACAAT
Cox8b	CCAGCCAAAACTCCCACTT	GAACCATGAAGCCAACGAC
Dhh	CACGTATCGGTCAAAGCTGA	TAGTTCCCTCAGCCCCTTC
Disp1	CTTCAGCAGGAGGGGAGAC	TGGCGATGTAATTCCCCAGG
Disp2	AGGGCCGAGGAAAGTGTG	GCTCCTGTTCTATGCGACAC
Fasn	TAGAGGGAGCCAGAGAGACG	TTGGCCCAGAACTCCTGTAG
Fu	TGCCTCTCAGCCTTCTTAGG	TAAGAGCGCCCCATACCA
Gli1	Qiagen QuantiTect Primer Assay	
Gli2	Qiagen QuantiTect Primer Assay	
Gli3	Qiagen QuantiTect Primer Assay	
Hhat	CTGGGAGTCACTGTGGAGAG	TGAGCATGGAGGTAGAGCAG
Ihh	GCTCACCCCCAACTACAATC	GCGGCCCTCATAGTGTAAAG
Mpzl2	TGTGCTTCCACTTCTCCTGA	TCCACAGCTTCTGTAGGACAAA
Pdk4	TGTGGTCCCTACAATGGCTC	CCACATCACAGTTTGGGTCG
Ppara	CGTACGGCAATGGCTTTATC	TCATCTGGATGGTTGCTCTG
Pparg	ATGGAAGACCACTCGCATTC	GCTTTATCCCCACAGACTCG
Ppargc1a	GGACAGAATTGAGAGACCGC	CGTCCACAAAAGTACAGCTCG
Prdm16	CGAGAAGTTCTGCGTGGATG	AGGCACCTTCTTTCACATGC
Ptch1	ACTCCAAAAGAAGAAGGCGC	CCAGAAGCAGTCCAAAGGTG
Ptch2	CCGAGTGGCTGTAATTGAGAC	CTGGAGGTGCAAGTCAAGTG
Shh	TCCAAAGCTCACATCCACTG	CTCCGGGACGTAAGTCCTTC
Smo	GCAAGCTCGTGCTCTGGT	GGGCATGTAGACAGCACACA
Srebf1a	CAGACACTGGCCGAGATG	AAACAGGCCCGGGAAGTC
Srebf1c	GAGCCATGGATTGCACATTTG	AGGCCAGAGAAGCAGAAGAG
Sufu	CTTCCAGTCAGAGAACACCT	TTGGGCTGAATGTAACTCCT
Tmem26	TGGTGTGGACGTGGAGTATG	GGTTTGGGGCTTTCTGGATG
Ucp1	GGCCTCTACGACTCAGTCCA	TAAGCCGGCTGAGATCTTGT
Ywhaz	TTACTTGGCCGAGGTTGCT	TGCTGTGACTGGTCCACAAT

Table S2. Significantly changed lipid species in SAT adipose tissue from female Hh-KO compared to Hh-WT mice.

lipid specie	fold change	log2(FC)	raw p value
TG 40:1:0	0.33381	-1.5829	0.00020198
PE 44:7:0	0.29431	-1.7646	0.00044111
TG 36:0:0	0.29036	-1.7841	0.00050384
TG 38:1:0	0.31787	-1.6535	0.00050828
DG 36:1:0	0.27978	-1.8376	0.0010552
CE 18:1:0	0.27607	-1.8569	0.0010989
PE 42:7:0	0.36157	-1.4676	0.0011177
PE O- 34:2:0	0.23029	-2.1185	0.0022397
TG 36:1:0	0.42910	-1.2206	0.0025228
PC 38:4:0	0.30720	-1.7028	0.0025786
SM 34:1:2	0.42812	-1.2239	0.0032551
TG 50:1:0	0.39347	-1.34570	0.004175

SM 36:1:2	0.40076	-1.31920	0.0043404
SM 34:1:3	0.54313	-0.88063	0.0045952
PE O- 36:2:0	0.16177	-2.62800	0.0047516
PE 36:1:0	0.29140	-1.77900	0.0053997
PC O- 36:5:0	0.25307	-1.98240	0.0063187
LPC 18:0:0	0.32003	-1.64370	0.0065327
PS 36:1:0	0.22738	-2.13680	0.0069129
TG 52:2:0	0.45124	-1.14800	0.0069496
TG 37:0:0	0.44343	-1.17320	0.0071863
LPC 20:4:0	0.21589	-2.21160	0.007924
SM 36:2:2	0.48070	-1.05680	0.0079363
PS 38:0:0	0.37886	-1.40030	0.0088746
TG 40:2:0	0.47644	-1.06960	0.0089333
PE 42:8:0	0.48996	-1.02930	0.011893
PC 33:2:0	0.23583	-2.08420	0.01198
PE O- 38:5:0	0.32608	-1.61670	0.012397
PE O- 36:5:0	0.37068	-1.43180	0.01245
PE O- 38:6:0	0.31739	-1.65570	0.01246
PE O- 36:3:0	0.21505	-2.21730	0.014042
SM 42:2:2	0.47781	-1.06550	0.014905
PC 32:0:0	0.40096	-1.31850	0.014992
TG 54:3:0	0.45635	-1.13180	0.015523
PC 34:1:0	0.37739	-1.40590	0.017115
PC 35:1:0	0.40279	-1.31190	0.01787
PE 36:2:0	0.45637	-1.13170	0.018181
DG 42:8:0	0.44271	-1.17560	0.018631
TG 37:1:0	0.54092	-0.88651	0.020191
PC 38:5:0	0.45307	-1.14220	0.020414
PS 38:4:0	0.45643	-1.13150	0.022402
TG 49:1:0	0.57035	-0.81007	0.02431
TG 38:2:0	0.46614	-1.10120	0.024563
PE O- 38:7:0	0.46850	-1.09390	0.024896
PE O- 40:7:0	0.41930	-1.25390	0.025291
PC 40:6:0	0.50034	-0.99901	0.025932
PE O- 39:5:0	0.38866	-1.36340	0.026357
CE 18:2:0	0.39014	-1.35790	0.029123
LPC 16:0:0	0.44701	-1.16160	0.029677
PC 36:4:0	0.45146	-1.14730	0.032026
PC 40:4:0	0.40860	-1.29120	0.033343
LPE 18:0:0	0.39997	-1.32200	0.034068
PC 35:5:0	0.46864	-1.09350	0.03444
PE 34:1:0	0.50525	-0.98492	0.036002
TG 46:0:0	0.45735	-1.12860	0.036087
DG 34:2:0	0.57057	-0.80952	0.037021
PC 36:2:0	0.51431	-0.95928	0.037114
PE 40:4:0	0.46985	-1.08970	0.039845
PE O- 40:6:0	0.46579	-1.10230	0.040253
TG 53:2:0	0.56188	-0.83168	0.042248

TG 48:4:0	2.19660	1.13530	0.042911
PC O- 34:1:0	0.57579	-0.79638	0.043363
LPE 20:4:0	0.52970	-0.91676	0.043673
LPE 18:1:0	0.33459	-1.57950	0.045743
TG 60:1:0	0.51717	-0.95130	0.046117
PC O- 38:11:0	0.49283	-1.02080	0.046615
PS 38:3:0	0.56647	-0.81994	0.049472
SM 42:1:2	0.49105	-1.02610	0.049661

Table S3. Significantly changed lipid species in SAT adipose tissue from male Hh-KO compared to Hh-WT mice.

lipid specie	fold change	log2(FC)	raw p value
TG 44:5:0	1.80040	0.84835	0.024026
TG 46:5:0	1.90170	0.92731	0.033319
TG 44:4:0	1.68070	0.74904	0.048773
DG 33:1:0	0.34448	-1.53750	0.04983

Table S4. Significantly changed lipid species in VAT adipose tissue from female Hh-KO compared to Hh-WT mice.

lipid specie	fold change	log2(FC)	raw p value
TAG [60:2]	0.028521	-5.13180	7.06E-05
PA [38:4]	0.22572	-2.14740	9.93E-05
LPC [18:1]	0.38589	-1.37370	0.00011788
LPE [18:1]	0.40238	-1.31340	0.00059117
PC [34:1]	0.40000	-1.32190	0.00067996
LPC [18:0]	0.409810	-1.28700	0.00091694
PG [37:5]	0.013574	-6.20300	0.0016002
PA [36:2]	0.139780	-2.83880	0.0018955
LPE [18:0]	0.37024	-1.43350	0.0021976
LPC [16:1]	0.50522	-0.98503	0.0022691
PA [40:6]	0.25376	-1.97850	0.0024683
LPC [16:0]	0.53103	-0.91314	0.0025391
PG [34:1]	0.41550	-1.26710	0.0027286
SM [36:1]	0.57208	-0.80572	0.0027715
PI [38:4]	0.49416	-1.01690	0.0028313
LPE [18:2]	0.47408	-1.07680	0.0034458
DAG [38:6]	18.17900	4.18420	0.0035224
PG [36:2]	0.38710	-1.36920	0.0035892
SM [36:2]	0.54645	-0.87183	0.0036618
DAG [34:4]	4.48260	2.16430	0.0040137
LPC [20:4]	0.45186	-1.14610	0.0048770
LPC [18:2]	0.48933	-1.03110	0.0052756
DAG [36:5]	3.94510	1.98010	0.0053088
LPC [17:0]	0.29604	-1.75620	0.0055779
DAG [36:4]	5.14430	2.36300	0.0067521
DAG [34:3]	3.70390	1.88910	0.0085195
TAG [52:6]	3.33590	1.73810	0.0086328
LPE [20:4]	0.52293	-0.93532	0.0087184

DAG [36:1]	0.66606	-0.58628	0.0112960
DAG [32:2]	3.10960	1.63670	0.012049
PG [40:7]	0.32414	-1.62530	0.01215
TAG [50:5]	3.02320	1.59610	0.012509
TAG [50:4]	2.91000	1.54100	0.013925
TAG [48:3]	2.61080	1.38450	0.015026
DAG [36:3]	3.53970	1.82360	0.016909
PG [38:6]	0.28134	-1.82960	0.017459
TAG [54:7]	2.83500	1.50340	0.017504
TAG [46:2]	2.10410	1.07320	0.018382
DAG [32:1]	2.43690	1.28500	0.019569
TAG [49:3]	2.61590	1.38730	0.020826
LPC [22:6]	0.25287	-1.98350	0.021971
PC [36:2]	0.58721	-0.76804	0.022001
PC [36:5]	0.58202	-0.78085	0.022041
TAG [52:5]	2.68220	1.42340	0.022795
DAG [38:5]	4.14200	2.05030	0.025174
PG [36:1]	0.31309	-1.67540	0.026177
TAG [46:3]	2.321000	1.21470	0.028177
TAG [46:1]	1.69570	0.76185	0.028514
PG [36:3]	0.53125	-0.91254	0.028768
TAG [48:2]	1.88310	0.913100	0.02926
TAG [50:3]	2.13440	1.09380	0.029971
TAG [56:8]	4.80390	2.26420	0.031937
TAG [44:1]	1.63480	0.70912	0.033004
PC [32:0]	0.63522	-0.65468	0.033217
TAG [48:4]	2.42220	1.27630	0.034962
LPE [16:0]	0.61977	-0.69019	0.035763
PC [40:6]	0.53614	-0.89933	0.039578
DAG [34:2]	2.15380	1.10690	0.040815
TAG [51:4]	2.41720	1.27330	0.041785
PC [32:2]	0.55483	-0.84987	0.044931
DAG [32:0]	1.80600	0.85280	0.047110
PC [38:4]	0.55043	-0.86138	0.047216

Table S5. Significantly changed lipid species in VAT adipose tissue from male Hh-KO compared to Hh-WT mice.

lipid specie	fold change	log2(FC)	raw p value
PG [40:7]	10.34700	3.37110	0.0030337
PS [38:5]	0.57389	-0.80115	0.016384
PS [40:4]	0.46698	-1.09860	0.022656
PS [36:1]	0.10478	-3.25460	0.038168
PG [34:1]	3.24250	1.69710	0.039762

Table S6. Numeric values of expression analysis of browning-related genes in (**A**) female and (**B**) male Hh mice adipose tissue quantified by qPCR shown in Figure 4A as heatmap.

Table S6A. Female Hh mice

gene name	tissue	Hh-WT		Hh-KO	
		mean	SD	mean	SD
Ucp1	BAT	1262.796	712.070	1514.575	664.074
	SAT	0.024	0.020	14.040	15.068
	VAT	1.820	1.578	26.950	31.449
Cox8b	BAT	54.859	29.015	36.949	15.797
	SAT	0.529	0.329	1.850	1.521
	VAT	0.987	0.571	2.440	1.715
Prdm16	BAT	6.912	2.831	6.058	2.652
	SAT	12.041	11.587	7.411	4.892
	VAT	1.131	0.670	5.884	3.032
Ppara	BAT	31.223	9.755	67.229	18.206
	SAT	0.183	0.156	4.917	5.949
	VAT	1.109	0.751	7.448	9.132
Mpzl2	BAT	7.180	2.837	8.734	3.572
	SAT	1.108	0.681	1.176	0.804
	VAT	3.332	2.509	2.466	1.097
Tmem26	BAT	5.111	3.587	5.038	3.757
	SAT	24.439	20.409	3.367	3.282
	VAT	22.023	9.788	9.541	4.082

Table S6B. Male Hh mice

gene name	tissue	Hh-WT		Hh-KO	
		mean	SD	mean	SD
Ucp1	BAT	418.970	269.136	506.378	199.057
	SAT	5.970	3.419	31.018	49.667
	VAT	5.081	7.918	10.866	17.045
Cox8b	BAT	8.710	1.628	6.104	1.563
	SAT	0.278	0.164	0.336	0.505
	VAT	0.007	0.002	0.040	0.049
Prdm16	BAT	15.710	7.365	7.513	1.830
	SAT	3.729	0.914	4.155	1.748
	VAT	2.229	0.967	7.524	7.767
Ppara	BAT	92.563	29.853	131.459	8.807
	SAT	5.287	3.050	3.957	4.479
	VAT	2.777	0.763	3.475	3.818
Mpzl2	BAT	1.186	0.159	0.747	0.317
	SAT	0.200	0.124	0.045	0.027
	VAT	0.158	0.053	0.144	0.131
Tmem26	BAT	3.415	3.423	3.419	2.805
	SAT	44.894	28.173	18.683	8.070
	VAT	9.229	4.848	35.514	24.352

Table S7. Numeric values of expression analysis of lipid synthesis-related genes in (**A**) female and (**B**) male SAC adipose tissue quantified by qPCR shown in Figure 4B as heatmap.

Table S7A. Female Hh mice

gene name	tissue	Hh-WT		Hh-KO	
		mean	SD	mean	SD
Fasn	BAT	0.25519	0.03738	0.27796	0.08601
	SAT	0.04512	0.03395	0.16481	0.06059
	VAT	0.13667	0.08534	0.20400	0.12671
Agpat	BAT	0.00083	0.00013	0.00103	0.00019
	SAT	0.00129	0.00075	0.00197	0.00083
	VAT	0.00415	0.00362	0.00244	0.00153
Pdk4	BAT	0.40141	0.09857	0.65438	0.23361
	SAT	0.02916	0.01041	0.10675	0.08653
	VAT	0.09025	0.04992	0.14615	0.07763
Srebf1a	BAT	0.00112	0.00058	0.00079	0.00046
	SAT	0.00015	0.00017	0.00047	0.00015
	VAT	0.00068	0.00048	0.00095	0.00082
Srebf1c	BAT	0.01971	0.00602	0.01873	0.00854
	SAT	0.00357	0.00184	0.00947	0.00591
	VAT	0.01358	0.01292	0.00969	0.00643
Ppargc1a	BAT	0.12619	0.12783	0.04135	0.01597
	SAT	0.05278	0.02282	0.02735	0.00431
	VAT	0.03014	0.01669	0.02030	0.00274

Table S7B. Male Hh mice

gene name	tissue	Hh-WT		Hh-KO	
		mean	SD	mean	SD
Fasn	BAT	3.79897	1.28528	2.48848	0.98842
	SAT	0.48474	0.12379	0.14522	0.08801
	VAT	0.21453	0.15567	0.15534	0.14725
Agpat	BAT	0.00106	0.00032	0.00078	0.00061
	SAT	0.00167	0.00054	0.00137	0.00096
	VAT	0.00484	0.00192	0.00072	0.00031
Pdk4	BAT	0.71462	0.33234	1.43578	0.87648
	SAT	0.05902	0.02926	0.06867	0.04303
	VAT	0.04390	0.04362	0.12122	0.08036
Srebf1a	BAT	0.00875	0.00531	0.00813	0.00596
	SAT	0.00196	0.00014	0.00116	0.00081
	VAT	0.00279	0.00138	0.00264	0.00109
Srebf1c	BAT	0.06093	0.03793	0.02828	0.01682
	SAT	0.00560	0.00423	0.00237	0.00236
	VAT	0.01303	0.00543	0.00620	0.00379
Ppargc1a	BAT	0.10433	0.07613	0.07736	0.09534
	SAT	0.00663	0.00276	0.00150	0.00074
	VAT	0.00567	0.00161	0.00510	0.00199

Table S8. Numeric values of expression analysis of Hedgehog-related genes in (**A**) female and (**B**) male SAC adipose tissue quantified by qPCR shown in Figure S6 as heatmap.

Table S8A. Female Hh mice

gene name	tissue	Hh-WT		Hh-KO	
		mean	SD	mean	SD
Ptch1	BAT	0.03790	0.01556	0.04321	0.01700
	SAT	0.00715	0.00266	0.01684	0.00371
	VAT	0.02004	0.00551	0.01984	0.01117
Ptch2	BAT	0.00400	0.00142	0.00335	0.00160
	SAT	0.00236	0.00110	0.01122	0.00649
	VAT	0.00878	0.00252	0.01414	0.01117
Ihh	BAT	0.00055	0.00050	0.00047	0.00038
	SAT	0.00175	0.00214	0.00156	0.00118
	VAT	0.00069	0.00041	0.00123	0.00063
Shh	BAT	0.00005	0.00004	0.00003	0.00003
	SAT	0.00016	0.00012	0.00010	0.00011
	VAT	0.00011	0.00009	0.00026	0.00028
Dhh	BAT	0.00076	0.00040	0.00054	0.00050
	SAT	0.00111	0.00142	0.00032	0.00024
	VAT	0.00017	0.00005	0.00020	0.00009
Disp1	BAT	0.02369	0.01035	0.02688	0.01203
	SAT	0.00396	0.00099	0.00744	0.00178
	VAT	0.01322	0.00429	0.01680	0.01254
Disp2	BAT	0.00026	0.00016	0.00139	0.00304
	SAT	0.00046	0.00054	0.00065	0.00034
	VAT	0.00062	0.00024	0.00103	0.00086
Hhat	BAT	0.00224	0.00078	0.00178	0.00083
	SAT	0.00280	0.00277	0.00223	0.00116
	VAT	0.00362	0.00128	0.00264	0.00118
Smo	BAT	0.16542	0.08355	0.10829	0.05673
	SAT	0.16501	0.08193	0.16530	0.09195
	VAT	0.33338	0.09595	0.22280	0.10940
Fu	BAT	0.00201	0.00079	0.00356	0.00398
	SAT	0.00183	0.00215	0.00245	0.00167
	VAT	0.00173	0.00112	0.00229	0.00104
Sufu	BAT	0.01678	0.00521	0.02033	0.00660
	SAT	0.00643	0.00420	0.00866	0.00241
	VAT	0.00908	0.00187	0.01261	0.00463
Gli1	BAT	0.24822	0.32424	0.17591	0.10338
	SAT	0.26155	0.30188	0.48731	0.28160
	VAT	0.26669	0.13445	0.54032	0.18560
Gli2	BAT	0.05296	0.01615	0.03480	0.01296
	SAT	0.02621	0.01181	0.05245	0.00924
	VAT	0.06339	0.01376	0.13700	0.05662
Gli3	BAT	1.19060	0.94325	0.62026	0.18718
	SAT	2.77699	3.03925	1.95884	0.96843
	VAT	0.92247	0.59301	3.39159	2.70223

Table S8B. Male Hh mice

gene name	tissue	Hh-WT		Hh-KO	
		**mean**	**SD**	**mean**	**SD**
Ptch1	BAT	0.02230	0.01016	0.01858	0.00924
	SAT	0.00628	0.00320	0.00799	0.00510
	VAT	0.01283	0.00407	0.03279	0.01657
Ptch2	BAT	0.00126	0.00106	0.00115	0.00065
	SAT	0.00189	0.00112	0.00191	0.00117
	VAT	0.00282	0.00134	0.00921	0.00788
Ihh	BAT	0.00017	0.00015	0.00008	0.00001
	SAT	0.00016	0.00012	0.00019	0.00017
	VAT	0.00061	0.00054	0.00114	0.00106
Shh	BAT	0.00008	0.00009	0.00011	0.00007
	SAT	0.00051	0.00068	0.00028	0.00022
	VAT	0.00075	0.00060	0.00247	0.00220
Dhh	BAT	0.00046	0.00024	0.00041	0.00025
	SAT	0.00037	0.00013	0.00024	0.00016
	VAT	0.00022	0.00007	0.00060	0.00036
Disp1	BAT	0.00549	0.00327	0.00573	0.00252
	SAT	0.00164	0.00095	0.00150	0.00144
	VAT	0.00180	0.00076	0.00670	0.00434
Disp2	BAT	0.00154	0.00069	0.00068	0.00054
	SAT	0.00201	0.00091	0.00107	0.00056
	VAT	0.00179	0.00084	0.00306	0.00301
Hhat	BAT	0.00394	0.00204	0.00208	0.00107
	SAT	0.00231	0.00100	0.00191	0.00139
	VAT	0.00297	0.00101	0.00346	0.00079
Smo	BAT	0.06473	0.01504	0.06518	0.03451
	SAT	0.04761	0.00950	0.04744	0.01850
	VAT	0.05346	0.02139	0.17248	0.12004
Fu	BAT	0.00083	0.00046	0.00045	0.00032
	SAT	0.00024	0.00012	0.00017	0.00005
	VAT	0.00029	0.00010	0.00154	0.00201
Sufu	BAT	0.01770	0.00490	0.01348	0.00461
	SAT	0.00411	0.00226	0.00362	0.00205
	VAT	0.00652	0.00119	0.01140	0.00295
Gli1	BAT	0.00053	0.00051	0.00167	0.00202
	SAT	0.00042	0.00039	0.00066	0.00052
	VAT	0.00056	0.00027	0.00412	0.00446
Gli2	BAT	0.00234	0.00129	0.00235	0.00183
	SAT	0.00119	0.00047	0.00277	0.00164
	VAT	0.00587	0.00128	0.00718	0.00617
Gli3	BAT	0.08375	0.01146	0.12537	0.11324
	SAT	0.16536	0.11603	0.16043	0.09279
	VAT	0.14195	0.11451	0.41022	0.26150

Table S9. Binding sites of GLI1 in hepatocytes of male C57BL/6N mice identified by ChIP-seq associated to FGF21 signaling.

chromo-some	start	end	length	peak summit	peak val	avg val	genes	dist to start	position
2	94.157.949	94.158.349	400	94.158.096	84	65	Hsd17b12	-187	upstream
2	157.737.324	157.737.573	249	157.737.344	27	22	Ctnnbl1	-57	upstream
4	118.437.253	118.437.607	354	118.437.440	81	54	Elovl1, Cdc20, Mpl	9347, -97, 20055	downstream, up-stream, downstream
4	148.448.303	148.448.663	360	148.448.384	48	37	Ubiad1, Mtor	-3633, -198	upstream, upstream
4	148.558.214	148.558.791	577	148.558.432	171	102	Mtor, Exosc10	109850, 5	downstream, in gene
4	107.879.610	107.879.882	272	107.879.680	65	53	Lrp8, Magoh	77421, -133	downstream, up-stream
5	114.942.244	114.942.454	210	114.942.272	23	19	Oasl1, 2210016L21Rik, Hnf1a	19032, 70, 28790	downstream, in gene, downstream
7	113.207.050	113.207.403	353	113.207.232	130	91	Arntl	-233	upstream
10	85.127.971	85.128.209	238	85.128.128	37	32	Mterfd3, Cry1	-101, 56926	upstream, down-stream
10	128.696.203	128.696.756	553	128.696.448	160	100	Rab5b, Cdk2, Pmel	-180, 8603, -9810	upstream, down-stream, upstream
11	95.041.357	95.041.579	222	95.041.472	54	47	Pdk2, Itga3	-101, 35242	upstream, down-stream
11	69.094.970	69.095.194	224	69.095.072	63	54	Vamp2, Per1	6544, -3884	downstream, up-stream
11	69.098.778	69.099.097	319	69.098.880	58	48	Vamp2, Per1	10352, -76	downstream, up-stream
11	60.210.320	60.210.626	306	60.210.448	84	62	Srebf1	10.156	in gene
11	60.222.732	60.223.008	276	60.222.848	75	60	Srebf1, Tom1l2	-2244, 130057	upstream, down-stream
11	98.192.945	98.193.603	658	98.193.312	188	96	Med1, Cdk12	-19, -9993	upstream, upstream
11	98.202.156	98.203.225	1.069	98.202.656	424	179	Med1, Cdk12	-9363, -649	upstream, upstream
13	96.670.931	96.671.177	246	96.671.008	35	30	Hmgcr	-72	upstream
13	119.689.917	119.690.510	593	119.690.176	71	49	1700074H08Rik, Hmgcs1	10134, -286	downstream, up-stream
14	18.238.150	18.238.454	304	18.238.304	66	52	Nr1d2	802	in gene
14	18.238.548	18.239.031	483	18.238.688	46	35	Nr1d2	418	in gene
15	6.708.312	6.708.534	222	6.708.448	31	28	Rictor	67	in gene
15	98.762.840	98.763.546	706	98.763.232	283	136	Arf3, Wnt10b	-114, 14918	upstream, down-stream
15	39.006.241	39.006.487	246	39.006.368	34	29	Fzd6	88	in gene
16	38.088.775	38.089.285	510	38.088.992	103	70	BC031361, Gsk3b	268, -9	in gene, upstream
18	34.330.734	34.331.247	513	34.331.008	118	69	Apc, Srp19	110024, -137	downstream, up-stream
19	46.395.115	46.395.481	366	46.395.264	78	58	Actr1a, Sufu	471, -1654	in gene, upstream
19	3.575.556	3.576.065	509	3.575.872	50	40	Ppp6r3, Lrp5	-123, 110692	upstream, down-stream
